# A Socio-technical assessment of the success of picture archiving and communication systems: the radiology technologist’s perspective

**DOI:** 10.1186/1472-6947-13-109

**Published:** 2013-09-22

**Authors:** Wen-Sheng Tzeng, Kuang-Ming Kuo, Huang-Wei Lin, Tai-Yuan Chen

**Affiliations:** 1 Department of Medical Imaging, Chi-Mei Medical Center, No. 901, Zhonghua Rd., Yongkang Dist., 710 Tainan, Taiwan; 2 Department of Medical Imaging and Radiological Science, College of Health Sciences, Central Taiwan University of Science and Technology, Taichung, Taiwan; 3 Department of Healthcare Administration, I-Shou University, No.8, Yida Rd., Yanchao District, 82445 Kaohsiung, Taiwan

**Keywords:** Socio-technical evaluation, Information systems success model, Picture archiving and communication systems (PACS)

## Abstract

**Background:**

With the increasing prevalence of Picture Archiving and Communication Systems (PACS) in healthcare institutions, there is a growing need to measure their success. However, there is a lack of published literature emphasizing the technical and social factors underlying a successful PACS.

**Methods:**

An updated Information Systems Success Model was utilized by radiology technologists (RTs) to evaluate the success of PACS at a large medical center in Taiwan. A survey, consisting of 109 questionnaires, was analyzed by Structural Equation Modeling.

**Results:**

Socio-technical factors (including system quality, information quality, service quality, perceived usefulness, user satisfaction, and PACS dependence) were proven to be effective measures of PACS success. Although the relationship between service quality and perceived usefulness was not significant, other proposed relationships amongst the six measurement parameters of success were all confirmed.

**Conclusions:**

Managers have an obligation to improve the attributes of PACS. At the onset of its deployment, RTs will have formed their own subjective opinions with regards to its quality (system quality, information quality, and service quality). As these personal concepts are either refuted or reinforced based on personal experiences, RTs will become either satisfied or dissatisfied with PACS, based on their perception of its usefulness or lack of usefulness. A satisfied RT may play a pivotal role in the implementation of PACS in the future.

## Background

Information Systems (ISs) are adopted to improve service, quality of information, and organizational efficiency [1]. Since ISs usually involve considerable financial investment, the measurement of their success has become a critical issue in healthcare and non-healthcare industries [2-4].

The definition of a successful IS has become a topic of substantial research and debate [3-7]. Most researchers [8-10] agree that evaluation of an IS should focus on the organizational influences surrounding the IS. These influences may be viewed from numerous perspectives including the usefulness of an IS [11,12], its usage [13], the information quality produced by an IS [14], the value of the IS to the organization [8], and its return on investments [15]. Although a variety of measures are available to evaluate the success of an IS, no single measure is superior to another [3]. Often, the choice of measures is dependent on the type of IS being assessed [4].

### IS success model

#### DeLone and McLean’s (1992) IS success model

**Figure 1:**
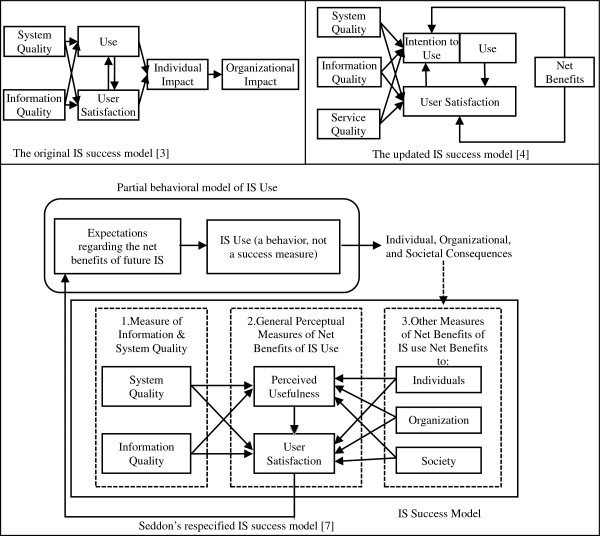
IS success models.

DeLone and McLean [3] proposed a classification system consisting of six measures of IS success drawn from a review of 180 studies performed to evaluate the success of ISs. The proposed IS success taxonomy included System Quality, Information Quality, IS Use, User Satisfaction, Individual Impact, and Organizational Impact. These variables are interrelated, as shown in Figure 1. Furthermore, Figure 1 demonstrates how system quality and information quality collectively influence both IS use and user satisfaction which, in turn, are determinants of individual impact. Finally, individual impact affects organizational impact.

In the DeLone and McLean model, IS use was regarded as an IS success variable. IS use was viewed as the consumption of IS output [3], which was considered to be an antecedent of individual impact. According to their model, IS use was indispensable for the realization of the system’s benefits. However, DeLone and McLean do not empirically validate their model but they recommend further development and corroboration of their proposed model [3].

#### Seddon’s (1997) model

Seddon [7] asserted that the merger of causal and process concepts in the IS success model proposed by DeLone and McLean [3] could become a source of confusion. Therefore, Seddon [7] proposed three classes of variables in his revised model (Figure 1). These included measures of information and system quality, general measures of net benefits from IS use, and behavior with respect to IS use. An important discrepancy between Seddon’s [7] model and that of DeLone and McLean’s [3] was the definition and placement of IS use. Seddon viewed IS use as a behavioral outcome that manifested as an anticipation of net benefits from utilizing an IS. This latter definition of IS use implied that IS use resulted from IS success, rather than being an innate feature of IS success [16]. Seddon’s [7] model included a direct path leading from system quality and information quality to perceived usefulness and user satisfaction. In addition, perceived usefulness was felt to influence user satisfaction.

#### DeLone and McLean’s (2003) updated IS success model

In 2003, DeLone and McLean [4] proposed an updated IS success model (Figure 1) and assessed its usefulness by considering vigorous changes in IS practice. They agreed with Seddon’s assertion that merging causal and process perspectives of IS success into a single model could be confounding. On the other hand, they argued that Seddon’s [7] restructuring of the DeLone and McLean [3] model into two partial causal models could confound the success model and deviate from the original intention of their model. Based on previous studies, DeLone and McLean [3] proposed an updated model by including service quality as a new dimension for measuring IS success, further assembling all the ‘impact’ measures into a single construct named net benefits.

### Picture archiving and communication systems (PACS)

With progress in image processing technology and network communications, the Picture Archiving and Communication Systems (PACS) has being increasingly utilized to process digitized medical images in healthcare institutions [11,17]. As a result, the PACS adoption rate among Taiwanese hospitals has risen from 24.9% in 2005 to 36% in 2013 [18,19]. PACS is an electronic IS used to acquire, store, transmit, and display medical images [20]. Proponents of PACS emphasize its numerous benefits such as the elimination of expensive silver-based films, reduction in physical storage requirements, improvement in access to images, and reduced personnel costs [9]. However, the implementation of PACS is a complex process which demands vast resources [21].

The adoption of PACS is dissimilar to other types of ISs [17] due to its innate complexity. For instance, PACS is required to integrate and process data from various hospital information systems (HIS) in order to provide sufficient patient information to the entire healthcare staff. Furthermore, the establishment of PACS is an expensive undertaking requiring justification for its cost [22]. Therefore, assessment of the success of PACS varies from that of traditional ISs.

When implementing new technologies, the existing literature [12,23,24] has emphasized the importance of focusing on wider organizational and human factors instead of centering solely on operational considerations. Technology, the individual, and the organization should not be assessed separately from each other [9]. Furthermore, Van Der Meijden et al. [25] concluded (after a thorough literature review) that more in-depth assessments of healthcare ISs be undertaken in order to explore a wider scope of factors that may determine the success or failure of these systems. The success of PACS requires integration/cooperation involving project management, financial and human resources, social/behavioral influences (e.g., change and resistance management), and technology (e.g., integration with various hospital information systems) [26].

In spite of its significant value to healthcare institutions, its widespread use, and the sizable financial investments involved, there is a paucity of literature emphasizing the social and technical factors involved in the successful utilization of PACS [26]. Most PACS-related studies have focused on evaluation of its effect on profit [8] or productivity [8,9,27]. Literature concerning the perceptions of PACS is still rare [11,12,17,26]. As shown in Table 1, most studies involved a variety of healthcare professionals including radiologists, technologists, and clinicians [11,12,26]. Moreover, the majority of studies have investigated the acceptance/adoption perception of PACS [11,12,17,28], and only one study [26] explored the success of PACS. Although such studies have advanced our knowledge regarding PACS, the use of a heterogeneous population may miss micro-level effects [29]. In addition, most PACS acceptance studies [11,12,28] utilized Davis’s [30] TAM or TAM-related models (e.g., UTAUT), probably the most commonly adopted model in healthcare settings [31], as a theoretical basis. Thus, additional studies, that are more homogeneous and that focus on the success of PACS, are required to gain a better perspective on the topic.

**Table 1:** Summary of perception-based studies of PACS

**Study type**	**Study**	**Population studies and setting**	**Analyzed sample size**	**Response rate**	**Variance explained**	**Theoretical base**
Adoption	Chang et al. [17]	Radiology department directors	35	53%	Not reported	Technology-Organization-Environment Model
Acceptance	Duyck et al. [28]	Radiologists and Technologists	56	59.6%	47-49%	Unified Theory of Acceptance and Use of Technology (UTAUT)
	Duyck et al. [12]	Radiologists and Physicians	Time1: 203	Not reported	Not reported	UTAUT
			Time2: 159			
	Aldosari [11]	Consultants, radiologists, residents, technologists, and others	89	74%	41%	Technology Acceptance Model
Evaluation of success	Paré et al. [26]	Radiologists (R), Technologists (T), and Clinicians (C)	232	27%	R: 79.2%	IS Success Model
					T: 58.7%	
					C: 64.1%	

The measurement of IS success, from the socio-technical perspective, should involve both social/human and technological factors [32]. As with most ISs, the success of PACS depends upon the degree of its utilization [11], which, in turn, may be directly related to system quality, information quality, service quality, user satisfaction, and perceived usefulness. Hence, the technological factors (i.e., system quality, information quality, and service quality) and the social/human factors (i.e., usefulness, user satisfaction, and PACS dependence) are practical constructs for measuring the success of PACS.

The motive for investigating our specific target population, i.e., radiology technologists (RTs), can be illustrated using the concept of the value chain [33]. A value chain can be regarded as a system of interdependent activities within an organization, including primary and supportive activities, which collectively generate value for the organization [33]. These activities are connected by linkages which exist when the manner in which one activity is performed affects the cost or effectiveness of the other activities [34]. Applying the concept of value chain to a radiology department, we can regard the radiologist’s interpretation of examinations as the primary activity and the RTs’ work as supportive activity. Without the RT’s ability to assess image quality via PACS, it is not possible for radiologists to interpret the images in a timely manner, which imposes a negative impact on patient care. Thus, the RT’s perception of PACS, as it relates to their jobs, is an important issue that hospitals must confront given the increasing rate of PACS adoption. Furthermore, literature that assesses the success of PACS from the perspective of non-physician healthcare providers [12,15,26,35] is relatively scarce.

The primary objective of this study was to propose (and validate) a revised PACS success model from the socio-technical perspective by quantifying the subjective perception of RTs. The motive for choosing this specific target population was twofold. First, the use of a more homogenous population would make it easier to capture micro-level effects [29]. Second, the concerns of RTs are important for gaining insight into how to promote wider PACS usage throughout the hospital.

## Methods

### Conceptual model formulation

DeLone and McLean [3] showed that abundant measures of IS success exist based on the different ways IS can be viewed. Consequently, the selection of success measures should rely upon the study context [16]. Most researchers agree that service quality, when well measured, can be coupled with system quality and information quality to assess IS success [36]. Furthermore, Seddon [7] asserted that ‘IS use’ was not an appropriate measure of IS success and felt that ‘perceived usefulness’ should be used, instead. However, other published literature [16] empirically affirmed that both DeLone and McLean’s [3] and Seddon’s [7] models exhibited reasonable fits. In other words, system quality, information quality, service quality, user satisfaction, and Seddon’s [7] perceived usefulness can all be regarded as valid measures of IS success.

According to DeLone and McLean [3], IS use as a measure of IS success makes sense for voluntary users only. Since PACS utilization is mandated in most healthcare institutions, PACS dependence (which measures the degree to which RTs are dependent on PACS for the execution of their tasks) is considered a more appropriate indicator of PACS success compared to DeLone and McLean’s [3] “use” or “intention to use”.

Paré et al. [26] adopted the IS success model to assess PACS success using radiologists, technologists, and clinicians as the study population. To the best of our knowledge, their study was the first of its kind to evaluate PACS success using the IS success model and it provided valuable insights into how to perform IS success studies. However, Paré et al. [26] did not test the full model with technologists because they felt that technologists interact with PACS in a more limited way. Thus, more in-depth investigations utilizing the perceptions of technologists are needed. Our study was based on the assertion that the full IS success model can be tested using RTs because its constructs, operational definitions, and measurements are properly adapted to the PACS context.

**Figure 2:**
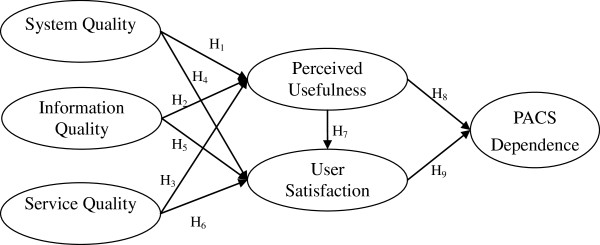
Research framework.

Adopting the viewpoint of DeLone and McLean [4], the proposed model was regarded as a causal model. The system quality, information quality, and service quality of PACS were pivotal in determining its perceived usefulness and RTs’ satisfaction with PACS. Furthermore, if the utilization of PACS enhanced the RTs’ job performance, they undoubtedly would experience a higher degree of satisfaction with the system and would depend upon it to a greater extent for their daily tasks. Finally, the RTs’ degree of satisfaction also influenced their dependence on PACS, as a greater extent of satisfaction would lead to an increasing dependence on PACS. The research framework justification, research variables, and their relationships in the proposed model are explained in detail in Figure 2.

This study adapted the variable definitions of prior IS success models [16,37] and modified them for use in the PACS context. The research framework was composed of the following six variables: system quality (in RTs’ PACS usage context, it measures the extent to which PACS is easy to use); information quality (refers to the extent to which the information quality of the output via PACS is sufficient, accurate, and up-to-date); service quality (represents the extent to which PACS performs the service correctly the first time and is able to fulfill its agreements); perceived usefulness (gauges the extent to which RTs believe that utilizing PACS has improved their job performance); user satisfaction (measures the degree of RT’s satisfaction with PACS); and PACS dependence (defined as the degree to which the RT is dependent on PACS for the execution of their tasks). The variables used in this study, their generic IS definition, and PACS-specific definition are shown in Table 2.

**Table 2:** Definitions of key constructs in this study

**Variables**	**Generic IS definition**	**PACS-specific definition**
System quality	The degree to which the SIS is easy to use [16].	The extent to which PACS is easy to use.
Information quality	The degree to which information produced has the attributes of content, accuracy, and format required by the user [16].	The extent to which the information quality of the output via PACS is sufficient, accurate, and up-to-date.
Service quality	The overall user assessment and service delivery assessment in the virtual marketplace [37].	The extent that PACS performs the service correctly the first time and the ability to fulfill its agreements.
Perceived usefulness	The degree to which the user believes that using a particular system has enhanced his or her job performance [16].	The extent that RTs believe that utilizing PACS has improved his/her job performance.
User satisfaction	The degree of user satisfaction with the system [16].	The degree of RT’s satisfaction with PACS.
PACS Dependence	The degree to which the user is dependent on the IS for the execution of their tasks [16].	The degree to which the RT is dependent on PACS for the execution of their tasks.

### Research hypotheses

#### The effect of system quality, information quality, and service quality on perceived usefulness

We aimed to study the effect of system quality, information quality, and service quality on the perceived usefulness of PACS. Since RTs are responsible for the correct entry of patient information into PACS and image storage [28], an easy-to-use PACS was anticipated. Furthermore, previous studies [38,39] argued that the users’ perception, that useful information would be produced by an IS, was related to the success of the system. Therefore, if the quality of information provided by PACS was deemed superior by RTs, they were more likely to perceive that PACS made a tangible contribution to their job performance. Thus, it was felt that information quality leads to the perceived usefulness of PACS. If the service quality of PACS is poor, the likelihood that RTs will perceive the system as useful will be diminished. If PACS is unreliable (e.g., prolonged downtime), RTs will experience a much lower level of perceived usefulness of the system. Previous literature [7,16,30,36,40,41] also supports positive relationships among system quality, information quality, and service quality, and perceived usefulness. Thus, the following three hypotheses were tested: H_1_: Higher levels of PACS system quality lead to higher levels of perceived usefulness of PACS; H_2_: Higher levels of PACS information quality lead to higher levels of perceived usefulness of PACS; H_3_: Higher levels of PACS service quality lead to higher levels of perceived usefulness of PACS.

#### The effect of system quality, information quality, and service quality on user satisfaction

DeLone and McLean [3] argued that system quality is an important antecedent to IT users’ satisfaction. A RT is unlikely to be satisfied if he/she has experienced problems in retrieving images or has had to wait a long time to acquire exam-related information via PACS. Furthermore, to carry out their tasks, RTs must acquire the most up-to-date and accurate information on the patients being examined. This implies that the search for patient examination information is one of the RTs’ most frequently cited reasons to use PACS. And it is common for RTs to use PACS to acquire the information they need. Regarding service quality, DeLone and McLean [3] argue that system quality and information quality may be the most important components for measuring a single system. In addition, most studies agree that service quality, when properly measured, can be lumped with system quality and information quality as constituents of IS success [36]. Besides, user satisfaction may be considered a response to users’ aspiration of service quality for a system [3]. That is, RTs subjectively assess the diverse consequences on a pleasant-unpleasant continuum after using PACS and formulate their own degree of satisfaction or dissatisfaction with PACS. Moreover, PACS can enhance RTs’ ability to obtain patient-related information, which leads to more accurate and comprehensive analyses of patient situations. Several prior studies [16,26,36,40,42-51] also suggest that system quality, information quality, service quality, and perceived usefulness are antecedents of user satisfaction. Thus, the following four additional hypotheses were tested: H_4_: Higher levels of PACS system quality lead to higher levels of user satisfaction with PACS; H_5_: Higher levels of PACS information quality lead to higher levels of user satisfaction with PACS; H_6_: Higher levels of PACS service quality lead to higher levels of user satisfaction with PACS; H_7_: Higher levels of perceived usefulness of PACS lead to higher levels of user satisfaction with PACS.

#### The effect of perceived usefulness and user satisfaction on PACS dependence

PACS may be the RT’s only viable choice for acquiring information pertaining to his or her task if no other means is available for accessing patient information. The more vital the information contained in an IS, the more the RTs will be forced to acquire the essential data via PACS. Furthermore, a key point of DeLone and McLean’s [3] model is that ‘IS use’ is regarded as an IS success measure. They defined ‘IS use’ as the utilization of IS output [3], which is viewed as an antecedent to individual impact. In other words, ‘IS use’ significantly influenced the realization of system benefits [16]. Furthermore, DeLone and McLean [3] argued that user satisfaction influenced ‘IS use’, as higher satisfaction led to greater dependence on an IS [16,49]. Since PACS utilization is mandatory in most healthcare institutions, it may be more appropriate to employ ‘dependence on PACS’ rather than ‘IS use’ to measure the success of PACS. Previous literature [16,49] also supports that perceived usefulness and user satisfaction are antecedents of IS dependence. Hence, the following two additional hypotheses were tested: H_8_: Higher levels of perceived usefulness of PACS lead to higher levels of dependence on PACS; H_9_: Higher levels of user satisfaction with PACS lead to higher levels of dependence on PACS. The proposed relationships among key constructs and supportive literature are summarized in Table 3.

**Table 3:** Proposed relationships among key constructs and supportive literature

**Relationships**	**Supportive literature**
System quality → Perceived usefulness	[16,30,36,40,45]
Information quality → Perceived usefulness	[36,44,48,52]
Service quality → Perceived usefulness	[36,52]
System quality → User satisfaction	[16,26,36,44,49,50]
Information quality → User satisfaction	[36,44,48,52]
Service quality → User satisfaction	[36,44,48,52]
Perceived usefulness → User satisfaction	[26,36,44,45,48,52]
Perceived usefulness → PACS dependence	[12,16,45]
User satisfaction → PACS dependence	[16]

### Study design

To assess the dependence of RTs on PACS, a cross-sectional survey was conducted at a large Taiwanese hospital. Information on the study hospital, study units, study users, tasks, processes, and policies regarding PACS are shown in Table 4. Questionnaires were used to collect the perceptions of RTs concerning PACS.

**Table 4:** Information on the study hospital, study units, study users, tasks, processes, and policies of PACS

**Study hospital**	
●	Academic, tertiary care, medical center including three campuses in southern Taiwan.
●	1335 beds; and more than 20,000 annual patient admissions in 2012.
●	Complete hospital-wide filmless operation of medical images with PACS.
**Study unit**	
●	Department of Medical Imaging. Including sections of abdominal imaging, neuroradiology, abdominal imaging, interventional radiology, thoracic and breast imaging, and nuclear medicine.
●	Totally 43 radiologists and 138 RTs.
●	The modalities that are provided for imaging diagnosis include traditional X-ray, computed tomography (CT) scan, magnetic resonance imaging (MRI), ultrasound, fluoroscopy, angiography, and gamma camera for nuclear medicine imaging.
**Study users of PACS**	
●	138 registered RTs who operate the aforementioned imaging modalities.
**PACS**	
●	The tasks and process of study users when operate PACS: Login the patient’s information into imaging modality, choose optimal imaging parameter, generate images, post-processing and quality control of the images, upload images to PACS, and recheck the images and associated information in PACS.
●	The policies of PACS: The PACS is implemented by INFINITT® with partnership strategy eight years ago and is upgraded to web-based version for three years.
●	The patient’s data of the PACS are supplied by a home-made HIS.

### Measures

The questionnaire used in this study consisted of two parts. The first part collected respondents’ demographic data, and the second part dealt with their perceptions regarding system quality, information quality, service quality, perceived usefulness, user satisfaction, and PACS dependence. In accordance with Churchill’s [53] approach to questionnaire generation, our study combined scales from other relevant empirical studies to generate an initial pool of items for each construct. Then, an expert panel comprised of one senior RT, one radiologist, and one researcher (who was specialized in healthcare information management) reviewed these items. Due to language and cultural differences, some of the items from the initial pool were deemed unsuitable and were dropped and others were modified according to suggestions from this panel of experts. System quality, information quality, service quality, perceived usefulness, user satisfaction, and PACS dependence were measured using existing instruments. With the exception of demographic questions, all questions listed were based on a 5-point Likert scale (1 for ‘strongly disagree’ and 5 for ‘strongly agree’).

The instruments for system quality utilized four items adapted from Teo et al. [54]. The information quality constructs were formed using four items from Rai et al. [16], Teo et al. [54], and Wang [36]. Service quality was measured in terms of four items adapted from Teo et al. [54] and Wang [36]. The instrument for perceived usefulness utilized four items based on Iivari [55]. User satisfaction was measured with a four-item scale developed by Chen and Cheng [44], Teo et al. [54] and Wang and Liao [50]. The instruments for PACS dependence utilized four items from Rai et al. [16] and Teo et al. [54].

A pilot test was conducted to develop these measures, followed by a broader survey. The pilot test was conducted using a convenient sample of 10 RTs, and items were subsequently modified resulting in a revised instrument that warranted further testing. Additional file 1 outlines the final measurement items.

### Sample and data collection

Permission from the Institutional Review Board of Chi-Mei Foundation Hospital, Taiwan, was acquired before proceeding with the investigation.

The research model was empirically validated using data collected from a survey. Questionnaires were distributed to 128 RTs in a large medical center including three campuses in southern Taiwan. The study population included every RT in the department of medical imaging listed in the subject hospital’s database, except those selected for the pilot test. From February 1^st^ to February 28^th^ 2013, subjects were asked to voluntarily fill out the paper-and-pencil survey. From the 128 RTs who comprised the study population, a total of 109 usable questionnaires were collected, representing a response rate of 85.16%.

## Results

### Descriptive statistics

Of the 109 respondents, 49.54% were male and 50.46% were female. More than 87% of the respondents were younger than 40 years of age. In addition, the majority were frontline RTs (88.07%). Most respondents had less than 5 years of work experience (40.37%). More than 68% of the respondents’ had between 1 and 10 years of job experience. Most respondents (68.81%) have 4–9 years of PACS experience, as shown in Table 5.

**Table 5:** Respondents’ characteristics

**Profile**	**Items**	**Frequency**	**Percentage**
**Gender**	Male	54	49.54%
	Female	55	50.46%
**Age**	≤ 30	45	41.29%
	31-40	50	45.87%
	41-50	12	11.01%
	≥ 51	2	1.83%
**Job title**	Head RT	13	11.93%
	RT	96	88.07%
**Current job experience**	≤ 5	44	40.37%
	6-10	31	28.44%
	11-15	12	11.01%
	≥ 16	22	20.18%
**PACS experience**	< 1	8	7.34%
	1-3	14	12.84%
	4-6	32	29.36%
	7-9	43	39.45%
	≥ 10	1	0.92%
	Others	11	10.09%

Furthermore, the majority of RTs’ perceptions about PACS were higher than 3 (based on a 5-point Likert scale), indicating that most RTs held positive perceptions toward PACS, as shown in Table 6.

**Table 6:** Reliability and validity

**Variable**	**Item**	**Mean**	**Standard deviation**	**Cross loading**	**CR**	**Cronbach’s α**	**AVE**
**System quality**	B1	3.95	0.63	0.86***	0.92	0.88	0.74
	B2	3.90	0.69	0.88***			
	B3	3.85	0.64	0.86***			
	B4	3.84	0.68	0.83***			
**Information quality**	C1	3.89	0.70	0.88***	0.94	0.91	0.79
	C2	3.88	0.68	0.91***			
	C3	3.91	0.67	0.89***			
	C4	3.92	0.67	0.88***			
**Service quality**	D1	3.83	0.71	0.86***	0.89	0.84	0.67
	D2	3.75	0.72	0.86***			
	D3	3.80	0.80	0.73***			
	D4	3.67	0.73	0.82***			
**Perceived usefulness**	E1	4.00	0.64	0.88***	0.91	0.87	0.71
	E2	3.83	0.66	0.78***			
	E3	3.91	0.70	0.88***			
	E4	4.04	0.65	0.84***			
**User satisfaction**	F1	3.83	0.62	0.86***	0.94	0.91	0.79
	F2	3.96	0.61	0.90***			
	F3	4.02	0.64	0.90***			
	F4	3.98	0.69	0.90***			
**PACS dependence**	G1	3.88	0.97	0.83***	0.92	0.88	0.74
	G2	4.00	0.67	0.86***			
	G3	4.23	0.70	0.86***			
	G4	4.17	0.69	0.89***			

Note: *CR* Composite reliability, *AVE* Average variance extracted.

*p < 0.05, **p < 0.01, ***p < 0.001.

### Data analysis

The proposed model and hypotheses were empirically validated using partial least square (PLS), a component-based structural equation modeling [56], supported by SmartPLS® 2.0 M3 software [57]. PLS was selected because of the small size of the samples collected [56]. A previous study [58] suggested a two-stage process for assessing the PLS model structure, including (1) the measurement model and (2) the structural model. The measurement model articulated the relationships between the latent variables and the measured (observed) variables, whereas the structural model articulated the relationships between the exogenous and endogenous latent variables [58].

#### Measurement model

The strength of the measurement model was demonstrated by calculating the reliability and validity (including convergent validity and discriminant validity) [58]. The present study conducted three tests [58] to determine the reliability of constructs in a single instrument including the reliability of items, composite reliability (CR), and Cronbach’s alpha of constructs. Reliability of the items was first gauged by inspecting the loading of each item on a corresponding construct. The results demonstrated reliability scores for all the items over the criterion of 0.707 [59]. Thus, the indicators measuring each construct in the present study all carried sufficient item reliability, as shown in Table 6. In the second step, PLS took into consideration the relationships among constructs when calculating the CR for each construct. The CR of all the constructs in the present study exceeded the criterion of 0.7 [58]. Additional evidence concerning the reliability of the constructs used in this study was acquired by computing Cronbach’s alpha. A value of 0.7 revealed sufficient construct reliability [58]. Based on these criteria, all constructs proposed were characterized by adequate reliability (Table 6). In the third step, PLS computed the average variance extracted (AVE) of each construct based on the degree to which corresponding indicators tapped into the same construct [56]. A figure of 0.5 demonstrated an adequate level of convergent validity for each construct [59] (Table 6).

Discriminant validity refers to the extent a given construct is different from other constructs [56]. As a rule of thumb, the square root of the AVE of each construct should be higher than the correlation of the specific construct with other constructs in the model [56] and should be at least 0.5 [59]. The results indicated that none of the inter-correlations of the constructs employed in the study exceeded the square root of the AVE for the construct, as shown in Table 7. Furthermore, the AVE scores of all constructs were larger than 0.5. All criteria demonstrated satisfactory discriminant validity of the model.

**Table 7:** Correlations among constructs

	**SQ**	**IQ**	**SeQ**	**PU**	**US**	**PD**
**System Quality (SQ)**	**0.86**					
**Information Quality (IQ)**	0.72	**0.89**				
**Service Quality (SeQ)**	0.66	0.77	**0.82**			
**Perceived Usefulness (PU)**	0.46	0.53	0.44	**0.85**		
**User Satisfaction (US)**	0.72	0.72	0.67	0.70	**0.89**	
**PACS Dependence (PD)**	0.64	0.61	0.50	0.68	0.70	**0.86**

Note: Diagonal elements show the square root of the average variance extracted (AVE).

#### Structural model

With sufficient measurement models, the hypotheses were then tested by inspecting the structural models. After calculating path estimates in the structural model, PLS utilized a bootstrapping technique to acquire the corresponding *t*-values. Hypothesis testing (resulting from the structural equation modeling analyses) are summarized in Table 8, in which the standardized path coefficients and *t*-values are shown. The results indicated that all nine hypotheses proposed in this study were significant, with the exception of H_5_.

**Table 8:** Structural model results

**Hypothesis**		***β***	**t-statistics**	**Supported?**
**H**_**1**_	System Quality → Perceived Usefulness	0.17	3.36***	Yes
**H**_**2**_	Information Quality → Perceived Usefulness	0.40	5.17***	Yes
**H**_**3**_	Service Quality → Perceived Usefulness	0.03	0.45	No
**H**_**4**_	System Quality → User Satisfaction	0.29	7.10***	Yes
**H**_**5**_	Information Quality → User Satisfaction	0.14	3.31***	Yes
**H**_**6**_	Service Quality → User Satisfaction	0.14	3.87***	Yes
**H**_**7**_	Perceived Usefulness → User Satisfaction	0.37	9.90***	Yes
**H**_**8**_	Perceived Usefulness → PACS Dependence	0.41	11.05***	Yes
**H**_**9**_	User Satisfaction → PACS Dependence	0.53	13.57***	Yes

*p < 0.05, **p < 0.01, ***p < 0.001.

**Figure 3:**
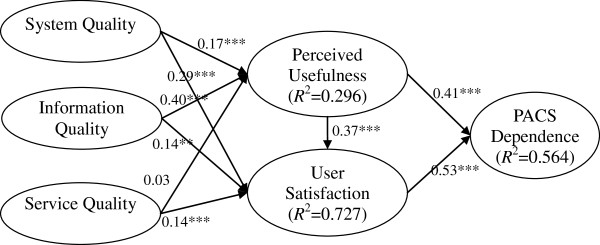
Structural model results.

As in the interpretation of multiple regression, the *R*^*2*^ indicates the amount of variance explained by the proposed model. The coefficient and *R*^*2*^ resulting from the PLS model are demonstrated in Figure 3. In terms of goodness of fit indicators, systems quality, information quality, and service quality accounted for 29.6% of the variance in perceived usefulness. Systems quality, information quality, service quality, and perceived usefulness accounted for 72.7% of the variance in user satisfaction. Perceived usefulness and user satisfaction accounted for 56.4% of the variance in PACS dependence. Furthermore, the global fit measure, GoF, was used to validate the PLS model and was calculated as $\left(\sqrt{\bar{\mathrm{AverageVarianceExtracted}\left(\mathrm{AVE}\right)}*\bar{R^{2}}}\right)$[60].

The average AVE = 0.74 and average *R*^2^ = 0.53, thus GoF = 0.63. According to Wetzels et al. [60], a GoF value of 0.63 (which exceeds the 0.36 threshold for large effect sizes) indicated that our model was valid [60].

The direct and total effect of user satisfaction on PACS dependence was 0.53. However, the total effect of perceived usefulness on PACS dependence was 0.60. Perceived usefulness, despite showing a weaker direct effect than user satisfaction on PACS dependence, demonstrated a stronger total effect on PACS dependence than that of user satisfaction. Among the three quality-related constructs, information quality had the strongest total effect (0.31) on PACS dependence. The direct, indirect, and total effects of system quality, information quality, service quality, perceived usefulness, and user satisfaction on PACS dependence are detailed in Table 9.

**Table 9:** The direct, indirect, and total effect of variables

	**Direct effect**			**Indirect effect**			**Total effect**		
	**PU**	**US**	**PD**	**PU**	**US**	**PD**	**PU**	**US**	**PD**
**SQ**	0.17	0.29			0.06	0.26	0.17	0.36	0.26
**IQ**	0.40	0.14			0.15	0.31	0.40	0.29	0.31
**SeQ**	0.03	0.14			0.01	0.09	0.03	0.15	0.09
**PU**		0.37	0.41			0.19		0.37	0.60
**US**			0.53						0.53

Note: *SQ* (System Quality), *IQ* (Information Quality), *SeQ* (Service Quality), *PU* (Perceived Usefulness), *US* (User Satisfaction), *PD* (PACS Dependence).

## Discussion

This study proposed and verified a PACS success model from a socio-technical perspective based on prior IS success models. All its constructs, including system quality, information quality, service quality, perceived usefulness, user satisfaction and PACS dependence, were proven to possess adequate psychometric properties and thus can be used as effective measures of PACS success. Although the relationship between service quality and perceived usefulness was not significant, other hypothesized relationships amongst the six measurement parameters of success were all confirmed. Furthermore, similar to prior studies, a socio-technical view [26,41] can be used to evaluate the success of an IS without placing sole emphasis on technology [61].

### The effect of system quality, information quality, and service quality on perceived usefulness

The results of hypothesis H_1_ supported the proposition that a positive relationship exists between system quality and perceived usefulness of PACS. Previous studies [16,30,36,40,45] also reflect similar results. Therefore, the perceived ease of use is a vital indicator of the RTs’ confidence in the ability of PACS to improve their work performance. Paré et al. [26] argued that users benefit from the adoption of PACS because the system removes the workload of handling physical films. Indeed, prompt access to medical images anywhere/anytime in the healthcare setting is the greatest benefit of all, and the inconvenience of lost or misplaced films [26] is no longer an issue.

Regarding hypothesis H_2_, results showed a positive relationship between information quality and perceived usefulness and were consistent with prior studies [36,41,52]. Indeed, by integrating PACS images with the patient’s relevant clinical information (e.g., present illness and allergic history) from other HISs, RTs are able to easily obtain the patient’s information during the examination process. The goals of PACS are the management and dissemination of patient images and relevant clinical information. Thus, it is incumbent on the RTs to acquire and utilize images as well as patient-related information from PACS.

In testing hypothesis H_3_, the results failed to support the proposition. These results differed from prior literature [36,52]. A plausible explanation might lie in the fact that although PACS provides the RT with obvious conveniences, the acquisition of patient images relies primarily upon the technical ability of the RTs, themselves [62]. Without sufficient know-how regarding basic radiographic techniques, it is not possible to acquire quality images even with PACS. Furthermore, during the non-PACS era, RTs did not need to upload images to PACS after examining the patients, a process which some RTs might regard as an extra burden. Therefore, despite the excellent services being provided by PACS, it is still possible that RTs consider such conveniences a burden.

### The effect of system quality, information quality, service quality, and perceived usefulness on user satisfaction

With regards to H_4_, the results support a positive correlation between system quality and user satisfaction with PACS and the results are consistent with prior studies [16,26,36,44,49,50]. By using PACS, RTs are able to execute their tasks in a single action because the required protocols for different exams are predefined. This represents one of the greatest advantages made possible by PACS.

The results of hypothesis H_5_ support the proposition that a positive relationship exists between information quality and user satisfaction. These results are in line with previous studies [36,44,48,52]. As DeLone and McLean [3] pointed out, user satisfaction might be regarded as the user’s anticipation of different properties of an IS: system quality, information quality, and service quality. The integration of HIS, Radiology Information Systems, and PACS enhances productivity as well as streamlines the acquisition of, and access to, patient information [8] such as subjective patient symptoms, objective clinical observations, diagnoses, and allergies. RTs are, therefore, likely to experience a greater degree of satisfaction with the comprehensive information provided by PACS.

In testing hypothesis H_6_, a positive relationship was found between service quality and user satisfaction. The results were in line with previous studies [36,44,48,52]. In the subject hospital, the PACS was outsourced to a software provider who was responsible for the PACS servers. The software provider was obligated to keep PACS in a non-stop working state due to the critical demands of healthcare. Overall, PACS has performed well in the subject hospital. RTs do not usually experience interruptions in their work caused by PACS downtime in the process of carrying out their routine tasks. This may explain why service quality is such an important factor in their expression of satisfaction with the system.

In testing hypothesis H_7_, results demonstrated a positive association between perceived usefulness and user satisfaction. These results indicated that usefulness plays a vital role in increasing the RT’s satisfaction with PACS. The results were consistent with the previous literature [26,36,44,45,48,52]. Indeed, the ability to easily produce and manage images is one of the greatest advantages when utilizing the system, which makes it possible to avoid the inconvenience of manually handling films. It is obvious that PACS reduces RTs’ workloads and increases in their degree of satisfaction.

### The effect of perceived usefulness and user satisfaction on PACS dependence

In testing hypothesis H_8_, results showed a positive relationship between perceived usefulness and PACS dependence. The results were consistent with prior studies [12,16,45]. Despite the mixed results regarding the influences of perceived usefulness and perceived ease of use on outcome behavior [63,64], our study was consistent with the findings related to the acceptance of technology in healthcare settings [65]. The use of the technology depends more on its usefulness than on its ease of use. It does not really matter how difficult PACS is to use; RTs will assimilate PACS into their routine if it is shown to be useful and helpful on the job and beneficial to patients. When PACS is already a part of their work procedure, convincing them to continue using the system is easier.

Regarding hypothesis H_9_, results showed a positive relationship between user satisfaction and PACS dependence and the results were consistent with prior studies [16]. The existing literature indicates that user satisfaction contributes to loyalty to a product/service [66,67] or dependence on an IS [16]. Thus, to further encourage the utilization of PACS, the hospital could enhance the RT’s satisfaction with PACS by strengthening its functions and versatility.

### A comparison of the results with previous studies

Compared with prior studies, Paré et al.’s [26] study accounted for 40.4% of the variance in net benefits, 60.8% of the variance in user satisfaction, and 48.5% of the variance in system continuance intention. Our model explained 29.6%, 72.7%, and 56.4% of the variance in perceived usefulness, user satisfaction, and PACS dependence, respectively. Furthermore, the “acceptance” is one of the measures that can be used to assess IS success [28]. Among these differing IS success measures, TAM and its related model (such as UTAUT) are the most commonly adopted theoretical bases in the literature [31]. We also compared our study with other studies which assessed PACS acceptance. These studies explained about 41%-49% of variance of PACS acceptance, as shown in Table 10.

**Table 10:** Comparison of model performance between TAM related models

**Study**	**Adopted model**	**Population**	**Analyzed samples**	**Response rate**	**Variance explained**
Duyck et al. [28]	UTAUT	Radiologists and technologists	56	59.57%	47%-49%
Aldosari [11]	TAM	Consultants, radiologists, residents, technologists, and others	89	74%	41%

## Conclusions

By modifying prior IS success models [3,4], we constructed a model to evaluate the success of PACS from a socio-technical perspective by substituting ‘use’ with ‘perceived usefulness’ [7] and adding PACS dependence to the equation. A survey of 109 RT questionnaires was used to test the hypotheses of the modified model. The model successfully predicted the dependence on PACS (*R*^2^ = 56.4%). However, our results contradicted the notion, derived from other studies, that service quality does not influence an RT’s perceived usefulness of PACS.

### Implications for research

The socio-technical model proposed in this study permitted simultaneous evaluation of the influences of system quality, information quality, and service quality, thus facilitating a more holistic approach to the evaluation of the success of PACS. In addition, most prior IS studies have focused on the associations among IS quality, satisfaction, and intention [44]. Furthermore, IS dependence, as one kind of system usage measure [16,68,69], has been less explored in settings where IS utilization is mandatory, especially in healthcare institutions. Therefore, the inclusion of the PACS dependence construct into the research framework provides a more complete framework for relevant constructs and their causal relationships. Finally, our model predicts user satisfaction better than PACS dependence. Thus, future studies could explore other dependent variables in order to obtain more knowledge on the topic of IS success.

### Implications for practice

Perceived usefulness and user satisfaction are vital to the success of PACS. Both were found to be significant antecedents to PACS dependence. Since perceived usefulness is the most important determinant of PACS dependence (total effect = 0.60), it is incumbent on managers to enhance the RTs’ dependence on PACS by strengthening their beliefs regarding how PACS can improve their performance and effectiveness.

In addition, perceived usefulness significantly mediates the effects imposed by system quality and information quality on PACS dependence. That is, the perceived usefulness of PACS reinforces user satisfaction, which, in turn, results in PACS dependence. The strategy for the target hospital is to increase perceived usefulness of PACS which can be developed by looking at its antecedents of system quality and information quality. This suggests that system quality and information quality must be provided to make RTs feel that PACS is helpful to them in their work routines.

Besides perceived usefulness, system quality is the second most important predictor of user satisfaction (total effect = 0.36). Consequently, enhancing the system quality increases the RTs’ satisfaction with PACS. Ease of use is the most important criterion of system quality. Thus, positive results regarding ease of use result in a positive impact on user satisfaction of PACS. Hospitals have an obligation to provide a system that is easy to use in order to enhance the RTs’ satisfaction. Furthermore it is also incumbent upon managers to improve the attributes of the system, such as ease of use, if it is to be more appealing to RTs.

Information quality is also a major factor affecting users’ satisfaction. Therefore, PACS should provide more relevant information to fulfill user requirements. This study suggests that when RTs felt that PACS was able to supply helpful information for carrying out their tasks, they are more likely to utilize it and experience an enhanced degree of satisfaction.

Most importantly, when RTs perceive that the information supplied by PACS is sufficient, accurate, and up-to-date for their tasks, they exhibit a higher degree of dependence on PACS. In addition, service quality also plays vital roles in the equation. Thus, managers should ensure that PACS provides reliable service with a superior user interface.

In conclusion, the findings of this study indicate that managers must improve the attributes of PACS because at the onset of deployment, an RT will establish his or her own personal perception regarding its quality (system quality, information quality, and service quality). When these perceptions are either reinforced or refuted, the RT will either be satisfied or dissatisfied with the system, and will also develop a perception of usefulness or lack of usefulness of PACS.

### Contributions and limitations

The results of the present study contribute to the field in a number of ways. In addition to advancing our knowledge regarding a method to evaluate the success of PACS, the results also aid in the understanding of the RTs’ dependence on PACS. In addition, our findings strongly support the appropriateness of the DeLone and McLean [3,4] model for understanding the determinants of success of PACS.

Our study had several limitations. The samples were collected in only one hospital within one country. Consequently, inferences to larger populations cannot be safely made. Future research should expand the present study’s findings by employing broader and more representative samples. Furthermore, a cross-cultural empirical study using a larger sample is essential for a more accurate generalization of the proposed model. In addition, although our model was based on the IS success model (a causal model asserted by DeLone and McLean), one must be careful when making such a causal declaration [70].

PACS is a new technology and generalization to other technologies is limited. Although our model predicted the success of PACS, other success measures such as benefits/return on investment, sustainability, global job satisfaction, and patient safety were not included in this study due to time and cost limitations. Due to the small sample size (n = 109), our results may suffer from insufficient statistical power. The definitions of constructs used in this study were adapted to RTs’ PACS usage context, which may differ from previous IS success studies. Further refinement of the operational definitions of constructs and measures is needed to better explain the constructs used. Our study used PACS dependence as a surrogate of system use which may not explain the variance completely. Although the relationship between user satisfaction and PACS dependence was confirmed in this study, there may be a bidirectional causal relationship between them. Further studies are needed to investigate the reciprocal relationship. Finally, our results may be limited by the depth of understanding permitted by closed-ended survey responses and the correspondence between self-reported perceptions and “reality.”

## Competing interests

The authors declare that they have no competing interests.

## Authors’ contributions

WS and KM conceived of this study and participated in its design, carried out the study, performed the statistical analysis, and drafted the manuscript. HW and TY helped to draft the manuscript and performed the statistical analysis. All authors read and approved the final manuscript.

## Pre-publication history

The pre-publication history for this paper can be accessed here:

http://www.biomedcentral.com/1472-6947/13/109/prepub

## Supplementary Material

Additional file 1 Questionnaire items. Click here for file
