# Supplementary material for: A Socio-technical assessment of the success of picture archiving and communication systems: the radiology technologist’s perspective
Source: BMC Med Inform Decis Mak. 2013 Sep 22;13:109. doi: 10.1186/1472-6947-13-109 (PMC3849362; doi:10.1186/1472-6947-13-109)
Supplement: Additional file 1 — Questionnaire items. [file 1472-6947-13-109-S1.doc]

Appendix 1. Questionnaire items

| Dimensions | Items |
| --- | --- |
| System Quality [54] | The adopted PACS in my hospital is easy to use |
|  | The adopted PACS in my hospital is user friendly |
|  | I find it easy to get PACS to do what I want it to do |
|  | Using PACS does not require a lot of effort |
| Information Quality [16,36,54] | PACS provides sufficient information to enable you to do your tasks |
|  | You are satisfied with the accuracy of PACS |
|  | The adopted PACS in my hospital provides up-to-date information |
|  | Through the adopted PACS in our hospital, I am able to access the information I need in time |
| Service Quality [36,54] | The adopted PACS in my hospital provides dependable services |
|  | The adopted PACS in my hospital gives prompt service to radiology technologists |
|  | The adopted PACS in my hospital is designed to satisfy the needs of radiologic technologists |
|  | You feel safe in your transactions with PACS |
| Perceived Usefulness [55] | Using PACS in my job enables me to accomplish task more quickly |
|  | Using PACS improves my job performance |
|  | Using PACS in my job increases my productivity |
|  | I find PACS useful at my job |
| User Satisfaction [44,50,54] | You are satisfied with PACS |
|  | PACS has met your expectations |
|  | I have a positive attitude toward PACS |
|  | PACS is effective in fulfilling your needs during the executing of examination tasks |
| PACS Dependence [16,54] | I am dependent on PACS to complete my task |
|  | I intend to continue using PACS rather than discontinue doing so |
|  | My intention is to continue using PACS rather than use any alternative means |
|  | I will not discontinue using PACS |
